# The impact of lower urinary tract symptoms on the quality of life during pregnancy: a cross-sectional study from Palestine

**DOI:** 10.1186/s12894-020-00761-9

**Published:** 2020-12-04

**Authors:** Qais T. Ahmad, Jaffar H. Saffarini, Ahmad M. Samara, Dima S. Jabri, Zaina H. Safarini, Yousra M. Banijaber, Ahmad Jaradat, Faris Abushamma, Sa’ed H. Zyoud

**Affiliations:** 1 Department of Medicine, College of Medicine and Health Sciences, An-Najah National University, Nablus, 44839 Palestine; 2 Department of Urology, An-Najah National University Hospital, Nablus, 44839 Palestine; 3 Department of Clinical and Community Pharmacy, Department of Pharmacy, College of Medicine and Health Sciences, An-Najah National University, Nablus, 44839 Palestine; 4 Poison Control and Drug Information Center (PCDIC), College of Medicine and Health Sciences, An-Najah National University, Nablus, 44839 Palestine; 5 Clinical Research Center, An-Najah National University Hospital, Nablus, 44839 Palestine

**Keywords:** Lower urinary tract symptoms, Quality of life, Pregnancy, Palestine

## Abstract

**Background:**

Lower urinary tract symptoms (LUTS) are prevalent among pregnant women. Several articles show the impact of LUTS on pregnant women’s quality of life (QoL). This study was designed to examine the impact of non-pathological LUTS on QoL among pregnant Palestinian women.

**Methods:**

A cross-sectional, hospital-based study was conducted on women who were pregnant and receiving regular antenatal care at the obstetrics and gynecology clinic in Rafidia Hospital, Palestine. This self-administered questionnaire included the Urinary Distress Inventory – short-form (UDI-6), the Incontinence Impact Questionnaire – short-form (IIQ-7), the European Quality of Life scale – 5 dimensions (EQ-5D), and the European Quality of Life – visual analogue scale (EQ-VAS). A convenience sampling method was used. In addition to this, multiple linear regression analyses were performed aiming to identify variables that have a significant relationship with QoL (i.e. socio-demographic variables, UDI-6 score, and IIQ-7 score).

**Results:**

This study included a total of 306 pregnant women. Participants had a mean age of 26.9 years (SD, 3.6). The subjects scored an average of 31.2 ± 19.2 out of 100 points for the UDI-6 scale and an average of 31.9 ± 24.9 out of 100 points for the IIQ-7 scale. On the other hand, the subjects’ average EQ-5D and EQ-VAS scores were 0.76 ± 0.17 and 67.96 ± 19.28 respectively. The subjects’ responses on UDI-6 significantly correlated with their responses on both the EQ-5D and EQ-VAS scales (r = − 0.338, *p* < 0.001 and r = − 0.206, *p* < 0.001, respectively). Likewise, their responses on IIQ-7 also significantly correlated with their responses on both the EQ-5D and EQ-VAS scales (r = − 0.389, *p* < 0.001 and r = − 0.329, *p* < 0.001, respectively). Regression analysis found that the UDI-6 score (*p* = 0.001) and IIQ-7 score (*p* < 0.001), were significantly and negatively associated with EQ-5D index scores.

**Conclusions:**

Our study shows a remarkable correlation between LUTS and QoL among pregnant women. Further longitudinal studies are required to assess the status of LUTS in the pre-pregnancy stage to ascertain a more accurate assessment of LUTS or LUTS related intervention and its impact on QoL during pregnancy.

## Background

Pregnancy is a physiological process that entails several multisystem changes [1]. Pathological conditions may appear during pregnancy such as Urinary tract infections (UTIs), pre-eclampsia, and gestational diabetes [2]. Few reports have been conducted to date to assess the impact of lower urinary tract symptoms (LUTS) during pregnancy [3–5]. LUTS can have negative effects on the social, physical, and emotional well-being of pregnant women [6]. Pregnancy-related LUTS, despite not being a pathological condition by definition, account for a significant amount of urinary distress and can affect quality of life (QoL) during pregnancy [7]. Moreover, the prevalence of urinary symptoms and distress during pregnancy could be under-reported due to the intimate nature of these symptoms [8].

QoL during pregnancy, with its effect on women’s physical and mental health, is an important topic that can be directly affected by the presence of LUTS [9–11]. Several reports have been published in order to identify and manage these symptoms early in pregnancy, such as antenatal urinary incontinence screening, health education regarding risk factors for urinary incontinence, and highlighting the importance of early recognition and management [7].

In Palestine, there has been a growing interest in studying QoL indices in different populations and how this may be impacted by different variables and factors [12–19]. However, the impact of LUTS on the QoL among pregnant women has not been examined before. Therefore, this study aimed to examine the impact of non-pathological LUTS on the overall QoL during pregnancy, and to identify factors that might directly affect the QoL. The merit of this study shadows the growing trend to integrate LUTS management into routine pregnancy-care, and planning personalised care for pregnant women in relation to these symptoms based on their unique risks and needs. Our results provide key data from the clinical setting demonstrating the extent to which LUTS affects pregnant women’s physical and psychological well-being. This in turn allows for better planning and establishment of management protocols for the problem in order to improve pregnancy and health outcomes in general. Finally, the current study will provide a reference point for health officials in order to assess the efficiency of any future interventions to address this problem.

## Methods

### Study design

This was a cross-sectional study that assessed the impact of LUTS on QoL during pregnancy. It used questionnaire-based interviews to collect data from the study subjects.

### Study setting and study population

The survey took place at the obstetrics and gynecology clinic in Rafidia Hospital. The target population was pregnant women aged between 18 and 40 years. Data was collected between September 2019 and February 2020. The appointments were undertaken between 8 AM and 1 PM which are the official clinic hours allocated to conducting follow-up appointments for pregnant women in the studied clinic.

### Sample size and sampling method

During the study period, the approximate number of pregnant women visiting the obstetrics and gynecology clinic at Rafidia Hospital was 1100. To determine the sample size required for analysis this number was used as a reference. Using the Raosoft sample size calculator, a sample size of 285 was determined by setting the response distribution at 0.50, the error margin at 5%, and the confidence interval at 95%. The target sample size was increased to 306 participants in order to decrease erroneous outcomes and improve research reliability. The convenience sampling technique was used for participants’ selection.

### Inclusion and exclusion criteria

For inclusion in the study, participants were required to be female, pregnant, able to speak Arabic, aged between 18 and 40 years, and have no history of a psychiatric illness. They were also required to be visiting the obstetrics and gynecology clinic in Rafidia Hospital for a pregnancy-related reason. Patients who had an established diagnosis of a urogenital condition or history of urological surgery were excluded.

### Data collection tool

For data collection, a four-section, Arabic-language questionnaire was used. Section one asked for the participants’ demographics. The structured questionnaire used in this study was based on previously published studies [20–23]. The Urinary Distress Inventory – short version (UDI-6) and the Incontinence Impact Questionnaire - Short Form (IIQ-7), were included in sections two and three respectively, to assess lower urinary tract symptoms including incontinence [20–22]. In the final section, we included the European Quality of Life scale 5 dimensions (EQ-5D) and the European Quality of Life visual analogue scale (EQ-VAS) [23, 24]. Data for this research was obtained through self-administered questionnaires. Physicians were available at all times to answer questions and explain any queries.

Section one asked the participants to fill in their age, weight, height, residency type, smoking status, employment status, education level, income, regular physical exercise, gestational trimester, parity (number of children), presence of psychiatric illness, medical disorders, long-term medication use, and previous surgeries. These factors were selected from those identified in previous studies that may affect QoL during pregnancy [25, 26]. Participants were put into one of five age groups: ≤ 20, 21–25 years, 26–30 years, 31–34 years, or ≥ 35 years [27]. The body mass index (BMI) for pregnancy was measured as pre-pregnancy weight in kilograms divided by height in meters squared, on the basis of self-reported weight and height. Based on their calculated body mass index (BMI), participants were classified into one of four groups: obese group (BMI ≥30 kg/m^2^), overweight group (BMI = 25–29.9 kg/m^2^), normal-weight group (BMI = 18.5–25 kg/m^2^), or the underweight group (BMI < 18.5 kg/m^2^) [28].

In section two, we included the UDI-6 – short version. Similar to its full version, UDI-6 was designed to assess the severity of urinary distress symptoms based on the level of discomfort experienced during the past month. UDI-6 contains six questions that cover three areas: irritative symptoms (items one and two), stress symptoms (items three and four), and obstructive or discomfort symptoms (items five and six). Participants answer each section by choosing one of four options: ‘greatly’, ‘moderately’, ‘a little bit’, and ‘not at all’. Each answer received a number of points between zero and three, with ‘greatly’ receiving three points and ‘not at all’ receiving zero points. Therefore, the highest possible UDI score was 18. The internal consistency of UDI, as previously tested by Cronbach’s alpha test coefficient, was 0.720. Permission to include the Arabic version of this tool in our study was granted by the developer [21].

In section three, we included IIQ-7 – short version. IIQ-7 is a tool designed to gauge the impact of urinary incontinence on life quality in women. As in its full version, IIQ-7 focuses on four areas: physical activity (items one and two), travel (items three and four), social relations (item five), and emotional wellbeing (items six and seven). The participant rated the severity of symptoms on a zero-to-three scale, with zero as the least severe, and three as the most severe [22]. Therefore, the highest possible IIQ-7 score was 21. The internal consistency of this tool was previously tested and the reported Cronbach’s alpha coefficient was 0.894. We obtained approval to include the Arabic version of this tool in our study from its developer.

All scores for UDI-6 and IIQ-7 were converted to a scale of 0–100 to allow comparison across measures [29]. UDI-6 and IIQ-7 proved to be valid and reliable questionnaires for evaluating the subjective phases of urinary incontinence severity. They were helpful in characterising the severity of incontinence, accessing treatment effectiveness, and making treatment decisions [30, 31].

In section four, we included the EQ-5D and the EQ-VAS scales to evaluate the health status and QoL of our subjects. EQ-5D is a standardized tool used to assess health outcomes. It studies five separate aspects of health: anxiety/depression, pain/discomfort, self-care, mobility, and usual activities. The subject rates each section on a 5-level scale (no problems, slight problems, moderate problems, severe problems and extreme problems). There are 3125 possible health states, obtained by using one number from each section, ranging from 11,111 (full health) to 55,555 (worst health). In the current study, we included the Arabic version [12, 15–19, 23] of EQ-5D in accordance with its developers’ guidelines after receiving their approval to use it (ID: 35675). The EQ-5D score was calculated according to the United States General Population Score Algorithm (i.e. EQ-5D-5L Crosswalk Index Value Calculator) for measuring the index value by the value set (weights) [32]. The possible values of this algorithm ranged from − 0.109 to 1, with values below 0 representing states considered to be worse than death. The internal consistency for this tool was acceptable (Cronbach’s alpha of 0.808). EQ-VAS, on the other hand, measures the subject’s perspective of their life quality using a scale of 0–100 points [23, 24].

### Statistical analysis

We used IBM SPSS, version 21 for data analysis. We presented the data as frequencies and percentages for participants’ characteristics, and as means and standard deviations for their questionnaire scores. Participants’ scores on EQ-5D and EQ-VAS scales were presented as medians and interquartile ranges. We tested the variables’ normality using the Kolmogorov-Smirnov test. Mann-Whitney and Kruskal-Wallis tests were implemented to test the contrast between different categories based on the participants’ characteristics. Possible correlations between all scales were evaluated by Pearson correlation coefficients. The significance was assumed at *p*-value < 0.05. We also carried out multiple linear regressions to predict the variables that had a significant relationship with QoL (EQ-5D and EQ-VAS scores as dependent variables). In our regression model, all variables with *p* < 0.05 in univariate analysis were used. Multicollinearity between the selected independent variables was tested with a variance inflation factor (VIF). Any VIF values of < 3 were considered to be appropriate due to a lack of collinearity.

## Results

### Demographic and clinical characteristics

A total of 306 participants took part in this study. Participants had a mean age of 26.9 years (SD, 3.6). The age and BMI categories which included the highest number of subjects were 21–30 years and normal BMI range, (70.2, and 46.1%, respectively). The majority of participants were educated to a university-level (71.2%). Most subjects were housewives (77.8%), and lived in a moderate-income household (48%). Over one-third of our subjects (41.2%) were pregnant in their third trimester. Only 8.2% had an established diagnosis of a chronic illness, and 10.1% practiced physical exercise regularly. Tables 1 and 2 summarise all demographic and clinical characteristics of the subjects.

**Table 1:** Association between the characteristics of the participants and the EQ-5D scores they achieved

Characteristic	No. (%); Total = 306	EQ-5D score Median [Q1-Q3]	***P***-value*
**Age group (years)**			
**≤** 20	22 (7.2)	0.79 [0.68–0.82]	0.323 ^a^
21–25	102 (33.3)	0.79 [0.67–0.86]	
26–30	113 (36.9)	0.79 [0.65–0.88]	
31–35	39 (12.7)	0.74 [0.60–0.82]	
> 35	30 (9.8)	0.80 [0.67–0.86]	
**BMI group**			
Healthy weight	141 (46.1)	0.81 [0.69–0.88]	**0.021** ^**a**^
Overweight	118 (38.6)	0.77 [0.65–0.83]	
Obese	47 (15.4)	0.74 [0.60–0.83]	
**Education level**			
Elementary school	4 (1.3)	0.76 [0.63–0.81]	**0.005** ^**a**^
Middle school	18 (5.9)	0.79 [0.71–0.84]	
High school	66 (21.6)	0.81 [0.71–1.00]	
University	218 (71.2)	0.77 [0.64–0.83]	
**Employment status**			
House-wife	238 (77.8)	0.70 [0.53–0.85]	0.133 ^a^
Governmental employee	31 (10.1)	0.65 [0.50–0.80]	
Private sector	37 (12.1)	0.70 [0.57–0.82]	
**Income**			
Low (< 2000 NIS)	123 (40.2)	0.79 [0.64–0.86]	0.577 ^a^
Moderate (2000–4999 NIS)	147 (48)	0.79 [0.68–0.84]	
High (≥5000 NIS)	36 (11.8)	0.80 [0.66–0.88]	
**Gestational trimester**			
First trimester	68 (22.2)	0.80 [0.70–0.87]	0.055 ^a^
Second trimester	112 (36.6)	0.80 [0.68–0.87]	
Third trimester	126 (41.2)	0.75 [0.63–0.83]	
**No. of children**			
0	60 (19.6)	0.79 [0.67–0.82]	0.869 ^a^
1	92 (30.1)	0.79 [0.67–0.86]	
2	64 (20.9)	0.80 [0.63–0.96]	
3	44 (14.4)	0.77 [0.65–0.84]	
≥ 4	46 (15.0)	0.78 [0.68–0.86]	
**Presence of chronic diseases**			
No	281 (91.8)	0.79 [0.68–0.86]	0.154 ^b^
Yes	25 (8.2)	0.74 [0.63–0.83]	
**Drug history**			
No	287 (93.8)	0.79 [0.68–0.86]	
Yes	19 (6.2)	0.74 [0.66–0.83]	
			0.361 ^b^
**Regular exercise**			
No	275 (89.9)	0.79 [0.68–0.86]	0.401 ^b^
Yes	31 (10.1)	0.80 [0.63–0.83]	

*Abbreviations*: *BMI* body mass index, *EQ-5D* European Quality of Life scale 5 dimensions, *NIS* New Israeli Shekel (1 New Israeli Shekel = 0.29 US Dollar)

* All significant *p*-values are displayed in bold

^a^ Calculated by Kruskal–Wallis test

^b^ Calculated by Mann–Whitney U test

**Table 2:** Association between the characteristics of the participants and the EQ-VAS scores they achieved

Characteristic	No. (%); Total = 306	EQ-VAS score Median [Q1-Q3]	***P***-value*
**Age group (years)**			
≤ 20	22 (7.2)	82.00 [68.00–90.00]	0.260 ^a^
21–25	102 (33.3)	70.00 [50.00–80.00]	
26–30	113 (36.9)	70.00 [60.00–85.00]	
31–35	39 (12.7)	60.00 [45.00–80.00]	
> 35	30 (9.8)	72.50 [63.75–86.25]	
**BMI group**			
Healthy weight	141 (46.1)	70.00 [57.50–90.00]	**0.031** ^**a**^
Overweight	118 (38.6)	70.00 [55.00–80.00]	
Obese	47 (15.4)	65.00 [50.00–80.00]	
**Education level**			
Elementary school	4 (1.3)	37.50 [27.50–40.00]	**0.004** ^**a**^
Middle school	18 (5.9)	55.00 [48.50–80.00]	
High school	66 (21.6)	70.00 [50.00–81.25]	
University	218 (71.2)	70.00 [60.00–85.00]	
**Employment status**			
House-wife	238 (77.8)	70.00 [53.75–85.00]	0.279 ^a^
Governmental employee	31 (10.1)	65.00 [50.00–80.00]	
Private sector	37 (12.1)	70.00 [57.50–82.50]	
**Income**			
Low (< 2000 NIS)	123 (40.2)	65.00 [50.00–80.00]	**< 0.001** ^**a**^
Moderate (2000–4999 NIS)	147 (48)	70.00 [55.00–80.00]	
High (≥5000 NIS)	36 (11.8)	80.00 [70.00–90.00]	
**Gestational trimester**			
First trimester	68 (22.2)	70.00 [60.00–80.00]	0.152 ^a^
Second trimester	112 (36.6)	70.00 [60.00–85.00]	
Third trimester	126 (41.2)	70.00 [50.00–80.00]	
**No. of children**			
0	60 (19.6)	75.00 [60.00–90.00]	0.136 ^a^
1	92 (30.1)	70.00 [60.00–85.00]	
2	64 (20.9)	70.00 [55.00–80.00]	
3	44 (14.4)	70.00 [50.00–80.00]	
≥ 4	46 (15.0)	62.00 [50.00–80.00]	
**Presence of chronic diseases**			
No	281 (91.8)	70.00 [57.50–85.00]	**0.008** ^**b**^
Yes	25 (8.2)	60.00 [40.00–70.00]	
**Drug history**			
No	287 (93.8)	70.00 [55.00–85.00]	**0.033** ^**b**^
Yes	19 (6.2)	60.00 [40.00–70.00]	
**Regular exercise**			
No	275 (89.9)	70.00 [50.00–80.00]	0.532 ^b^
Yes	31 (10.1)	80.00 [70.00–90.00]	

*Abbreviations*: *BMI* body mass index, *EQ-VAS* European Quality of Life visual analogue scale, *NIS* New Israeli Shekel (1 New Israeli Shekel = 0.29 US Dollar)

* All significant *p*-values are displayed in bold

** We merged underweight and healthy weight groups as the former contained only 7 subjects

^a^ Calculated by Kruskal–Wallis test

^b^ Calculated by Mann–Whitney U test

### QoL assessment findings (EQ-5D and EQ-VAS scores)

The participants’ mean EQ-5D and EQ-VAS scores were 0.76 ± 0.17 and 67.96 ± 19.28, respectively. Table 1 shows the association between the characteristics of the participants and the scores achieved on the EQ-5D scale, whereas Table 2 presents the relationship between their characteristics and scores on the EQ-VAS scale. The participants’ level of education significantly affected their scores on both EQ-5D and EQ-VAS scales (*p*-values were 0.005 and 0.004, respectively), whereas their level of income and history of previous chronic disease significantly correlated only with their EQ-VAS scores (*p*-values were < 0.001 and 0.008, respectively).

### Urogenital symptoms’ correlation with the QoL in pregnant women

Regarding urinary distress observations, the subjects scored an average of 5.6 ± 3.4 out of 18 (31.2 ± 19.2 out of 100) points for the UDI-6 scale, and an average of 6.7 ± 5.2 out of 21 (31.9 ± 24.9 out of 100) points for the IIQ-7 scale. Table 3 presents the correlations between urogenital symptom scales and QoL scales in pregnant women. The participants’ responses on UDI significantly correlated with their responses on both the EQ-5D and EQ-VAS scales (r = − 0.338, *p* < 0.001 and r = − 0.206, *p*-value< 0.001, respectively). Likewise, their responses on IIQ-7 also significantly correlated with their responses on both the EQ-5D and EQ-VAS scales (r = − 0.389, *p* < 0.001 and r = − 0.329, *p* < 0.001, respectively). As expected, the participants’ responses on the EQ-5D scale and EQ-VAS scale also showed significant correlation (r = 0.329, *p* < 0.001).

**Table 3:** Correlation of urinary distress scales and quality of life scales among pregnant women

Scales	EQ-5D ***P***-value	Correlation	EQ-VAS ***P***-value	Correlation
UDI	< 0.001	− 0.338	< 0.001	− 0.206
IIQ-7	< 0.001	− 0.398	< 0.001	− 0.348
EQ-5D	–	–	< 0.001	0.329
EQ-VAS	< 0.001	0.329	–	–

*Abbreviations*: *EQ-VAS* European Quality of Life visual analogue scale, *EQ-5D* European Quality of Life scale 5 dimensions, *UDI* urinary distress inventory, *IIQ-7* incontinence impact questionnaire - short form

### Results of multiple linear regression analysis

A multiple linear regression analysis was constructed according to BMI, educational level, UDI-6 score, and IIQ-7 score. Multiple linear regression models that were estimated to explore associations with the EQ-5D index score found that the UDI-6 score (*p* = 0.001) and the IIQ-7 score (*p* < 0.001) significantly and negatively correlated with the EQ-5D index score. The factors significantly associated with the EQ-5D index score based on multiple linear regression findings are illustrated in Table 4. There was no evidence of multicollinearity between independent variables (VIF ranged from 1.037–1.261).

**Table 4:** Multiple linear regression analysis of the association of variables with EQ-5D scores

Model		Unstandardized Coefficients		Standardized Coefficients	t	***p***-value*	95.0% Confidence Interval for B		Collinearity Statistics
		B	Std. Error	Beta			Lower Bound	Upper Bound	VIF
**1**	**(Constant)**	1.045	0.058		17.962	0.000	0.931	1.160	
	**BMI**	−0.013	0.013	−0.052	− 0.966	0.335	− 0.038	0.013	1.108
	**Education level**	−0.037	0.014	−0.040	−.992	0.052	−0.064	0.010	1.037
	**UDI-6 score**	−0.002	0.001	−0.191	−3.334	**0.001**	−0.004	−0.001	1.261
	**IIQ-7 score**	−0.002	0.000	−0.330	−5.927	**0.000**	−0.003	−0.002	1.196

*BMI* body mass index, *VIF* variance inflation factor, *EQ-5D* European quality of life scale 5 dimensions, *UDI* urinary distress inventory, *IIQ* incontinence impact questionnaire

* All significant p-values are displayed in bold

The multiple linear regression analysis was performed by using the EQ-VAS score as a dependent variable after controlling for BMI, education level, income, presence of chronic disease, drug history, UDI-6 score, and IIQ-7 score (Table 5). This demonstrated that income (*p* value = 0.036) was significantly and positively associated with EQ-VAS scores, whilst IIQ-7 scores (*p* < 0.001) significantly and negatively correlated with EQ-VAS scores.

**Table 5:** Multiple linear regression analysis of the association between factors and EQ-VAS score

Model		Unstandardized Coefficients		Standardized Coefficients	t	***p***-value*	95.0% Confidence Interval for B		
		B	Std. Error	Beta			Lower Bound	Upper Bound	VIF
**1**	**(Constant)**	69.385	8.852		7.839	0.000	51.965	86.804	
	**BMI**	−0.669	1.508	−0.025	− 0.444	0.657	−3.637	2.298	1.140
	**Education level**	2.665	1.674	0.091	1.592	0.113	−0.630	5.960	1.162
	**Income**	3.373	1.604	0.116	2.102	**0.036**	0.215	6.530	1.089
	**Presence of chronic diseases**	−7.004	7.401	−0.100	−0.946	0.345	−21.569	7.562	2.962
	**Drug history**	1.251	8.509	0.016	0.147	0.883	−15.494	17.997	2.065
	**UDI-6 score**	−0.110	0.080	−0.082	−1.371	0.171	−0.269	0.048	1.285
	**IIQ-7 score**	−0.217	0.045	−0.280	−4.821	**0.000**	−0.306	−0.129	1.209

*BMI* body mass index, *EQ-VAS* European Quality of Life visual analogue scale, *UDI* urinary distress inventory, *IIQ* incontinence impact questionnaire, *VIF* variance inflation factor

* All significant *p*-values are displayed in bold

The factors significantly associated with the EQ-VAS score based on multiple linear regression findings are illustrated in Table 5. There was no evidence of multicollinearity between independent variables (VIF ranged from 1.089–2.962).

## Discussion

There is scanty data to describe the prevalence of LUTS during pregnancy and its negative effect on QoL, which we believe is prevalent in Palestine, and may negatively affect the physical and mental health of pregnant women. We used two assessment tools for urinary symptoms (UDI-6 and IIQ-7) and two life quality assessment tools (EQ-5D and EQ-VAS) in order to explore this problem. In addition to this, factors that may affect QoL have also been studied. It was appropriate to use a generalised questionnaire to determine the subject’s health-related quality of life (HRQoL) values [33–36], given that most pregnant women do not have a fatal illness [37]. As one of the most common means for assessing the overall health of the body, EQ-5D is a powerful generic tool, particularly in regards to evaluating symptoms of pain and anxiety/depression [38]. In addition, IIQ-7 is a seven-item questionnaire designed for the assessment of various QoL impairment areas [39]. The areas include social activities, moving far from home, mental wellbeing, household chores, entertainment activities, feelings of frustration, and physical recreation.

The mean EQ-5D in our study was 0.76 ± 0.17, whereas the mean EQ-VAS score was 67.96 ± 19.28. There was also a strong correlation between urinary distress symptoms assessment scores (UDI and IIQ-7) and QoL assessment scores (EQ-5D and EQ-VAS). Therefore our study confirms the hypothesis that LUTS during pregnancy has a detrimental effect on QoL, suggesting a negative impact on physical and mental health during pregnancy.

The participants’ scores on QoL assessment scales showed significant correlations with some demographic variables such as BMI, education level, income level, history of other chronic illnesses, and use of chronic medication. This is supported by the Singaporean Integrated Women’s Health Program findings which showed a notable association of BMI, parity, and education level with urinary distress symptoms and a higher likelihood of seeking medical care due to these symptoms [40].

Participants with higher socioeconomic status had a better QoL score, which is consistent with findings from other studies that revealed a significant association between socioeconomic status and urinary incontinence. Furthermore, this cohort of patients is more likely to seek medical advice and treatment in earlier stages [41]. LUTS is a newly evolving concept in urology, as well as a broad term that can be applied to many urological abnormalities, such as lower urinary tract infections, idiopathic overactive bladder symptoms, etc. In general, higher-income and educated females are more likely to lead a healthy lifestyle in regards to eating healthily and undertaking regular exercise, as well as being less exposed to anxiety and other mental health issues. All of these elements reduce the risk of developing LUTS symptoms [41, 42]. This is also applicable to pregnant females, particularly as patients with a higher income have easier access to primary healthcare, allowing prompt treatment of LUTS and any underlying pathology. This observation should be taken into consideration if a screening program for LUTS were to be initiated, as prioritising lower-educated and lower-income females would likely identify a higher proportion of patients requiring treatment. Omitting higher-income and educated females from such a screening program would likely be a cost-effective approach.

We also uncovered a significant correlation between the severity of the urinary distress symptoms (using both UDI-6 and IIQ-7 scales) and the subject’s QoL. This emphasises the bothersome nature of these symptoms. This observation is in concordance with an increasing understanding of the fundamental effect of urinary incontinence symptoms on quality of life, and the importance of surveying and managing these symptoms as early as possible [43]. Proper and timely intervention for these problems should be of particular importance, as due to the intimate nature of urinary distress symptoms, affected women often delay seeking medical help.

### Limitations and strengths

One of the limitations of this study was the cross-section design, which prevented us from interpreting the causality of significant associations in our results. Another limitation of this study is that it took place in one hospital, which may limit the generalisability of our data to all pregnant women in Palestine. However, this study had strengths as well, such as using two urinary distress assessment tools and two QoL tools that have proven validity, which attest to the accuracy of our assessment. The most fundamental limitation lies in the fact that we wanted to exclude any established diagnosis of lower urinary tract abnormalities, therefore our exclusion criteria mandates omission of subjects with any previously established diagnosis of urinary tract abnormalities or previous urological surgeries. Nevertheless, we do believe that a small percentage of pregnant females could have had overactive bladder symptoms prior to pregnancy which became more prominent or distressing during pregnancy. We did not go back and try to review all symptoms prior to pregnancy, as our goal was to assess the prevalence of LUTS during pregnancy and its impact on QoL. An additional question that was not addressed in this study was whether the effect on bladder function alters during different trimesters. Whilst we have the data available and could have undertaken sub-analysis, we felt the sample size too small to present viable conclusions. Therefore, we preferred to analyse the data as a whole, rather than sub-classifying it. Furthermore, the absence of control groups (i.e. pregnant versus non-pregnant) limits the interpretation of the disease burden on HRQoL.

Moreover, this was the first study to examine urinary distress symptoms’ impact on the QoL during pregnancy in Palestine. As our study focus was towards pregnancy-associated, non–pathological LUTS, all of our participants had received a first-trimester negative urine test, and were screened for fever symptoms and dysuria at the time of the questionnaire. Patients with pre-existing urogenital conditions and a history of urological surgery were also excluded, as mentioned previously. Understanding of Pregnancy-associated LUTS/ Pregnancy non-pathological LUTS/ LUTS during pregnancy is an evolving concept in the literature [44] and we took the first step in the Middle East to evaluate its presence, risk factors, and impact on QoL.

## Conclusions

There is a remarkable correlation between the severity of urinary distress symptoms and the QoL among pregnant women. Identification and characterisation of HRQoL-related factors in pregnant women with LUTS can potentially accelerate the development of diagnostic, preventive, and therapeutic strategies for the improvement of HRQoL in this population. Alerting healthcare providers to the presence of LUTS among pregnant women is recommended. Focusing on the prevention of risk factors for LUTS is necessary in order to minimise its detrimental effect on QoL. Further longitudinal studies are required to assess the status of LUTS in the pre-pregnancy stage and ascertain a more accurate assessment of LUTS and LUTS related intervention and its impact on QoL during pregnancy. Several strategies can be adapted to recognise and treat this problem from an early stage, such as community-based counselling for pregnant females and regular assessment of LUTS during different stages of pregnancy. Therefore, healthcare providers should consider the psychological effects of LUTS on pregnant women in order to raise and deal with possible evolving mental health issues through different trimesters.

## Data Availability

All datasets collected and analyzed in this survey will be available by the corresponding author upon any reasonable request.

## Abbreviations

UTIs: Urinary tract infections
LUTS: Lower urinary tract symptoms
QoL: Quality of life
HRQoL: Health-related quality of life
NNU: An-Najah National University
NIS: New Israeli Shekel
UI: Urinary incontinence
EQ-5D: European Quality of Life scale 5 dimensions
UDI: Urinary distress inventory
IIQ: Incontinence impact questionnaire
EQ-VAS: European Quality of Life visual analogue scale
OBGYN: Obstetrics and gynecology
IRB: Institutional Review Board
VIF: Variance inflation factor
